# Female Pediatrics Residency Program Directors: Position Length, Program Size, and Career Impact

**DOI:** 10.1089/whr.2024.0165

**Published:** 2025-04-24

**Authors:** Liam M. Courtney, Ankith G. Rao, Veronica Vuong, Avani Vaghela, Amanda Brooks, Gregg C. Lund

**Affiliations:** ^1^ Rowan-Virtua School of Osteopathic Medicine, Stratford, New Jersey, USA.; ^2^ Rocky Vista University College of Osteopathic Medicine, Colorado, Parker, Colorado, USA.; ^3^ Rocky Vista University College of Osteopathic Medicine, Southern Utah, Ivins, Utah, USA.; ^4^ Volunteer mentor, Park City, Utah, USA.

**Keywords:** medical education leadership, gender leadership disparities, medical leadership career development

## Abstract

**Background:**

Currently, the majority of pediatricians are female. However, it is unknown if that general representation is seen along the academic continuum. This study aimed to describe the sex distribution, position duration, and program size of pediatric residency program directors (PDs) and compare female PD representation to other roles along the academic continuum.

**Methods:**

Data from all 213 U.S. pediatric residency programs were obtained from publicly available sources. Female representation along the professional academic continuum is obtained from publicly available sources. PD position duration was divided into three groups: short (<5 years), medium (5–10 years), and long (>10 years). Program size was based on the total resident count: small (<31 residents), medium (31–60 residents), and large (<60 residents).

**Results:**

The majority of PDs, 153 (72%), were classified as female. Within each duration grouping, there were significantly more females than males. There was no significant difference in the within-sex distribution of duration grouping in the current PD position between female and male PDs. There were significantly more females than males in each program size group. The within-sex distribution of the program size grouping was not statistically different between female and male PDs. Females are less represented in academic positions with greater authority, such as pediatrics department chairs (33.3%) or medical school permanent deans (27%), compared to 72% of PDs.

**Conclusion:**

Females are well-represented as pediatric PDs, but underrepresented in medical school positions with greater authority. Understanding the reasons for this is needed to ensure diverse and representative medical school leadership.

## Introduction

Representative leadership goes beyond Marian Wright Edelman’s quote, “You can’t be what you can’t see.” Leaders act not only as role models but also as guides for trainees, helping them navigate obstacles specific to them.^1^ In pediatrics training, residency program directors (PDs) are the leaders charged with shaping and supporting their programs and trainees.^2^ In addition to providing general support for all residents in training, leaders with similar life experiences to their trainees can identify and understand specific obstacles that individual trainees may face and, when possible, change the environment that creates them. However, PDs must have adequate organizational authority to make changes to effectively support the residents.

This is especially important for individuals who are underrepresented in medicine, or individuals from a majority group who still face societal and social obstacles, such as women in pediatrics. Today, female residents experience bias and harassment more than their male counterparts.^3^ Even at the faculty level, women pediatrics faculty report more instances of bias due to their sex than men. Male faculty members do not see bias in the work environment or report events as often as female coworkers.^3^ As most pediatric residents are female, it is unclear whether the current makeup of pediatric PDs reflects the residents for whom they are responsible. Nor do we know how the female representation continues along the academic continuum including positions with more authority to change the training and care system.

PD role was selected because it is a foundational leadership position in the pediatric academic continuum and would be a first step. Characterizing this role would not only be important knowledge but could lay the groundwork for other studies for the other roles along the continuum, such as fellowship training Program Director, Clinical Medical Directors, and subspecialty Division Directors.

This study aimed to (1) describe the sex distribution of pediatric PDs, (2) compare program size and position duration in their PD role between female and male PDs, and (3) assess female representation of pediatric PDs when compared to other roles along the professional academic continuum (pediatrics residents, pediatrics faculty, pediatrics department chairs, and medical school deans).

## Methods

For all U.S. Pediatrics residency programs, the following data were extracted from the Accreditation Council for Graduate Medical Education (ACGME) Public Accreditation Data System:^4^ Program Director’s name, their initial appointment date, and the total no. of the program’s approved resident positions. The sex distribution of U.S. medical students was extracted from medical schools accredited by the American Osteopathic Association Commission on Osteopathic College Accreditation^5^ and the Liaison Committee on Medical Education (LCME).^6^ The sex distribution of pediatrics residents,^7^ pediatricians,^8^ as well as LCME, accredited U.S. medical school pediatrics faculty,^9^ pediatrics department chairs^10^, and medical school deans^11^ was acquired from publicly available sources. The most current data at the time of the project was utilized for each group: Medical Students 2022, Pediatric Resident Trainees 2021, Pediatric Physicians 2021, Pediatric Faculty 2022, Pediatric Program Directors 2023, Pediatric Department Chairs 2022, and Medical School Permanent Deans 2022. Owing to the constraints of available data, a binary construct, female and male, was used to characterize sex. PD sex was determined through a systematic search of official program websites, starting with the residency website and faculty listing specifically identifying pronoun usage. The search of publicly available websites continued until pronoun usage was identified. If pronouns were absent, a secondary search was conducted using articles related to PD.^12^

PD’s current position duration was calculated as the completed years from their initial appointment to when the data were accessed (January 22, 2023). PD duration was grouped into three-time frames of completed years: short (less than 5 years), medium (5–10 years), and long (>10 years). Program size was categorized into three groups of total resident count: small (less than 31 residents), medium (31–60 residents), and large (>60 residents).

The percentages of females and males in each duration group (short, medium, and long) were calculated to assess the within-position duration group. Similarly, within-program size groups were calculated as the percentage of female and male PD in each program size group.

To characterize the within-sex distribution of PD position duration, the percentage of total female PDs in short-, medium-, and long-term positions and the percentage of total male PDs in the same duration categories were calculated.

Residency programs consist of many training sites. These may cross different region types (rural, suburban, and urban), program site ownership (university, community, for-profit vs. not-for-profit), and religious organization affiliation, to name a few. Because there is no standardized methodology or taxonomy to describe these hybrid systems, we did not include them in the methodology.

Two-tailed, two-sample Z-tests were used to compare the male and female distributions of leadership duration and program size. Within-sex proportions were tested for each position duration (*i.e.,* % of total females vs. % of total males in the short-term duration group) and within each program size (*i.e.,* % of total females and % of total males in short-term position durations). Two-tailed, two-sample Z-tests were performed to compare the proportion of females in the pediatrics PD population to that of medical students, pediatrics residents, pediatricians, pediatrics faculty, pediatrics department chairs, and medical school deans. Significance for all tests was achieved with *p* values less than a type 1 error of 0.05.

## Results

Data from all 213 programs were analyzed. The majority of PDs, 153 (72%) were classified as female. Within each position duration group, there were significantly more females than males. There was no significant difference in the within-sex distribution of PD in short-, medium-, and long-term position durations between females and males. For position duration, 63% of the total female PDs were in their short-term positions versus 55% of the total males, 26% of the total female PDs were in their medium-term positions versus 27% of the total males, and 11% of the total female PDs were in their long-term positions versus 18% of the total males. The data of the female versus male PD duration in the current PD position are displayed in Table 1.

**Table 1.** Female Versus Male Program Directors by Position Duration

Program director duration (complete years)	Female *N* (% duration group)	Male *N* (% duration group)	Total *N*	
<5	97 (75)	33 (25)	130	S
5–10	40 (71)	16 (29)	56	S
>10	16 (59)	11 (41)	27	S

S, Significant *p* value less than 0.05.

Each program size group (small, medium, and large) had significantly more females than males. Similarly, the within-sex distribution of program size (small, medium, and large) was not statistically different between the female and male PDs. The proportion of total females in small programs was 33% versus 37% of total males, 39% of total females in medium-sized programs versus 33% of total males, and 28% of total females in large programs versus 30% of total males. The program-size data are presented in Table 2.

**Table 2.** Female Versus Male Program Directors by Program Size

Program size (approved resident positions)	Female *N* (% size group)	Male *N* (% size group)	Total *N*	
<31	51 (70)	22 (30)	73	S
31–60	60 (75)	20 (25)	80	S
>60	42 (70)	18 (30)	60	S

S, Significant *p* value less than 0.05.

The female representation of the PD role compared to other populations in the academic continuum is presented in Table 3. Of note, females are less represented in positions with greater authority, such as pediatrics department chairs and medical school permanent deans, compared to females with PDs.

**Table 3.** Proportion of Females in Various U.S. Medicine Populations

Population	Females *N* (% of total)	Total *N*	*p* value
Medical Students	54,818 (54.5%)	100,645	S
Pediatric Resident Trainees	9,476 (72.8%)	6,894	0.765
Pediatric Physicians	39,189 (65.0%)	60,252	S
Pediatric Faculty	15,263 (61.0%)	24,996	S
Pediatric Program Directors	153 (71.8%)	213	**—**
Pediatric Department Chairs	52 (33.3%)	156	S
Medical School Permanent Deans	38 (27.1%)	140	S

S, Statistical significance compared to Pediatric Program Directors.

## Discussion

Females represented the vast majority (72%) of all pediatric PDs. There were more women than men for all durations of PD leadership and residency program sizes. Female trainees, therefore, at least related to sex, are likely to have appropriate role models. This is important because while male PDs may theoretically support their female trainees as a group, they have not experienced the harassment and bias of females, and male pediatrics faculty do not see the harassment their female coworkers are experiencing.^3^ It would likely be more difficult for male faculty members who have not experienced and do not see workplace biases against females to appropriately support female trainees and advocate for environmental changes. In addition, female mentees with male mentors reported more sexual harassment than male mentees with female mentors.^13^

To be effective, even leaders who represent trainees and see their obstacles must have adequate authority to change the environment that creates and perpetuates the identified obstacles. As with any role in any organizational hierarchy, the PD has less authority than other roles in the continuum. For example, the PD must approve faculty for the training program but does not have the authority to hire sub-specialty faculty. This authority may be in the subspecialty Division Director. However, the Division Director does not have the authority to hire faculty outside their sub-specialty, an authority held by the Departmental Chair.

These roles with greater authority have a greater authority to change the environment the Pediatric residents are trained. This highlights the concern that fewer women are in the roles of pediatrics department chairs and medical school deans, and roles with greater authority. Similar results have been reported for Obstetrics & Gynecology.^14,15^ With such a significant majority of PDs being female, it raises the question of why this majority female role has not translated into a greater representation of females as chairs and deans. Three potential explanations seem plausible, and the reality may be a combination of these and other unrecognized factors.

It is plausible that a large group of female faculty members choose to follow a career path ending in the PD role and have no interest in leadership positions with greater authority. In this role, they have the opportunity to support, train, and transform the next generation of pediatricians. The role of PD is honorable and necessary for pediatrics to successfully survive. However, it is unknown whether those who choose this role have the support, counseling, and mentoring to have chosen it with a complete understanding of other options. Gottlieb describes occupational gender segregation, in which women are directed toward relationship-oriented specialties. Within pediatrics, they may be guided to roles such as PD that are very relationship-oriented rather than more stereotypically male roles with greater authority.^16^

Another possibility is that the PD role is not on the professional path to becoming either a pediatrics departmental chair or a dean. The path to becoming a pediatrics chair remains to be documented. This unknown raises the question of what career guidance these female faculty can provide in ascending the academic career continuum.

Finally, the most ominous explanation is if the PD role is along the path to chair or dean, and female PDs want to attain these roles but are obstructed in that career path. Implicit and explicit sexism in academic medicine may be adequate obstacles to explaining this possibility, but there are also additional potential societal factors. This includes societal roles and environmental obstacles that limit women from ascending professionally and the lack of support to limit the effect of these obstacles. Female physicians reported bearing most household maintenance roles compared to their partners.^17^ After having a child, this discrepancy widens even further.^17^ 29 percent of the females studied reported taking extended leave after having a child, 25% chose a different specialty, 47% reduced work hours, and 25% changed practice settings. Additionally, 47% of these females passed on career advancement due to family responsibilities.^17^ Except for actual childbearing, these decisions are societal rather than biological. The lack of appropriate support and accommodation for female physicians at home and at work exacerbates this. Claudia Goldin, PhD, received the 2023 Nobel Prize for Economics for her work in this field. Hopefully, this will stimulate more attention to these factors and support potential remedies.^18,19^

Unless resident selection is very narrowly limited, it is not logistically possible for every resident with various identities to be part of a program where they have representation in their PD. However, in a predominantly female specialty, a better understanding of whether there is appropriate female representation in Pediatric leadership and the etiology of any lack of representation stands out as one critical area in need of understanding.

This study was limited by the sex characterization methodology. Our characterization of sex was primarily determined by pronouns used to describe the PD. This method is unable to differentiate if the pronoun usage was describing the sex or gender of the PD. This may introduce errors if there is a large group of transgender individuals. In addition, this study focused on the sex of the PD. There are other characteristics of trainees, faculty, and leaders, in addition to sex, including race, ethnicity, gender identity, and disability, which contribute to bias, harassment, or lack of support experienced in the workplace. These other characteristics may have different influences individually, and the intersection of these characteristics may be more than additive, with different characteristics related to varying degrees of significance at the intersections.

Another limitation was the lack of a standardized methodology or taxonomy to describe programs with multiple settings and training affiliations. These include hybrid programs that may be located in different regional locations (rural, suburban, or urban), settings with different organizational affiliations (university or community-based training settings), or different site ownership (not-for-profit, for-profit, or religious affiliation.)

Additional data must be made available to fully understand the status and progress of diversity, inclusion, and equity in pediatrics. This would include better characterizations of individual data, including sex, race/ethnicity, sexual orientation, socioeconomic upbringing, *etc.* Future studies should consider these factors both independently and intersectionally. Further information about the academic rank of PDs and the career path to becoming a chair or dean would provide additional insights.

## Abbreviations Used

ACGME: Accreditation Council for Graduate Medical Education
LCME: Liaison Committee on Medical Education
PD: Pediatric residency program director
